# XDR-TB in South Africa: Revised Definition

**DOI:** 10.1371/journal.pmed.0040161

**Published:** 2007-04-24

**Authors:** Timothy H Holtz

The authors should be commended for a thoughtful and stimulating article [1]. However, we wish to clarify the historical record about the use of the term XDR-TB. The concept of XDR-TB as a distinct nosological entity was first developed at the Centers for Disease Control and Prevention (CDC) in March 2005 and introduced into public use in October 2005 at the 36th World Conference on Lung Health in Paris [2,3]. At that meeting, data on second-line drug resistance from a global survey of supranational TB reference laboratories conducted by CDC and the World Health Organization, as well as treatment outcomes of XDR-TB patients in Latvia, were first presented. Shortly thereafter, the cluster of TB deaths with resistance to second-line drugs in HIV-infected persons in KwaZulu-Natal was presented at the 13th Conference on Retroviruses and Opportunistic Infections in Denver in February 2006 [4]. The original definition for XDR-TB published in the *Morbidity and Mortality Weekly Report* in March 2006 [5] that they have used, however, was revised in October 2006 at an emergency meeting of the Global XDR-TB Task Force. The revised definition was published on November 3, 2006 in an MMWR notice to readers [6]. Currently, XDR-TB is defined as the occurrence of TB in persons whose Mycobacterium tuberculosis isolates are resistant to isoniazid and rifampin plus any fluoroquinolone and at least one of the three injectable second-line drugs (amikacin, kanamycin, capreomycin). The definition was revised because drug susceptibility testing to these drugs produces reliable and reproducible results, and is more accessible in resource-limited settings. In addition, patients meeting the revised definition have significantly poorer treatment outcomes. The new definition is important for those intending to conduct surveillance for XDR-TB in their setting.
